# Infant Management

**Published:** 1874-01

**Authors:** F. K. Bailey

**Affiliations:** Knoxville, Tennessee


### INFANT MANAGEMENT.

BY F. K. BAILEY, M.D., KNOXVILLE, TENNESSEE.

The management of infants from the moment of birth onwards, is a subject which interests every practitioner. The exact time when the duties of the obstetrician cease, and those of the physician commence, may be before or after the cord is tied, but the details connected with the proper caring for the new being before us, includes a judicious treatment or management of the funis. The child under favorable circumstances, will of course, cry lustily from the first emerging of the head. Generally, too, the cord will be found hard and knotty, beating with great force. Some authorities advise severing the cord at once, while others say, "Wait till it collapses and pulsation ceases."

My own practice for years has been to desist while pulsation is strong.

The child can expand the lungs as well, and probably better, when blood is flowing along the old channel. Again, there is less danger from hemorrhage if the cord is flabby and reduced to its least dimensions, and those who have of late contended strongly that it is unnecessary to tie the cord at all, may have acquired the notion from having never severed it while the least pulsation continues. Let that question be decided as it may, there is certainly less danger from any inattention on the part of a physician or midwife, to a certain tightness of the ligature, which is insisted upon by all writers and teachers. The safest way of management appears to be to wait a sufficient length of time, tie tightly so that slipping will be out of the question, and then give watchful attention to the dressing of the portion which must still linger a while, till nature causes a separation.

During the first years of my practice, I had occasion to treat an unhealed umbilicus. I do not remember why there was a failure to heal, but the child, a boy of two or three when first brought to my notice, required the daily dressing of an open sore about the navel, as large as a silver half dollar. No application appeared to "heal" it, and it was still open as he grew up.

Whether the boy became a man with such an encumbrance I know not. This case served as a warning, and in no case when it was possible to do so, did I afterwards fail to attend personally to "the dressing of the navel." The usual appliance recommended in the books, and well known to all, is the best that can be devised, and needs only to be insisted on and adopted. In country practice, when the medical attendant generally tarries until after the customary repast is in readiness, and eaten, then is ample opportunity for sitting by and assisting in the preparation of the little pieces of soft linen, which are to be the first in order of application, of the many vestments which are to clothe the little stranger. But often in the hurry of city practice, this dressing is left entirely to the nurse.

It will readily be seen that for some days, a decomposing mass is to remain in close proximity to a very susceptible and tender tissue. To obviate all ill effects of such a contact, carbolic acid is a most excellent local application, in weak solution with glycerine. Lard, oil of olives or any other nuctuous preparation is unnecessary, if the parts are kept moistened with a lotion as above. Not many months ago, I was called to see a child whose navel was reported in a bad condition. On inspection, there were found a complete nest of maggots, and no one could account for their presence. The band had been so snugly fitted that a fly could not possibly gain access to the parts. On examining the dish containing lard, nsed in dressing, minute maggots were seen. Lard, unless rendered from select portions of the leaf at a butcher's stall, is not a safe application to any abraded surface. Glycerine, diluted with an equal amount of carbolic acid solution, is far better in every respect.

With an intelligent watchfulness in the daily dressings, no trouble need attend the separation of the cord.

Before leaving this locality, we may well consider another troublesome contingency in connection with the umbilicus.

*Umbilical hernia* is often met with, and found sometimes very difficult of management. It is said by Guersant to be caused by want of proper attention in supporting the belly with the body bandage, so that the cries of the child will protrude the intestine. Feebleness is another reason stated.

The above causes are very common, but I have often seen, this accident to occur in strongly formed children, where the parietal tissues were firm, and crying was not unusually hard or continuous. In such cases the opening appears larger than commonly seeen, and it has occured to my mind that a very large cord will leave a correspondingly large opening, which will permit the intestine to escape. In as much as large and vigorous children have a large cord, this idea is plausible, besides the fact that strong children will cry more vigorously than feeble ones. A hemispherical pressure pad, of a size sufficient to more than fill the opening, fastened by means of adhesive strips passing nearly around the body, is the best *truss* in such cases. Considerable care is requisite in order that the strips do not cause irritation, and that they be reapplied every two or three days at longest.

I have seen cases in which the dressings could not be dispensed with, until the abdominal contents had outgrown the size of the opening, or the omentum become developed so as to form a plug.

In my case-book I find notes of a case which may have been reported in some other connection.

The hernial tumor was as large as a hen's egg, and had never been properly treated. I found the child (nearly 12 months old) in this condition while treating it for a disease of the lungs. I made as a truss, a compress of cork, cut into proper shape and size, which, on account of its lightness, could be worn without being oppressive.

The child died soon after from the lung affection. No hope of a radical cure can be entertained, when the tumor has attained so large a size, and a life of annoyance and peril only awaits such a subject.

The responsibility resting upon a medical man cannot be duly estimated, when we consider how easily a little neglect or improper management, will lead to such results.

There are other details of management, which, though enjoined by the standard authors, are often neglected by the young practitioner, and perhaps older. One is putting the child to the breast as soon as the condition of the mother will admit. Dewees urges it for the reason that an infant may lose the *faculty* of sucking. I have in many instances known young mothers to be terribly afflicted with a mammary abscess, in consequence of an inability to make the child nurse, when the instinct is gone forever.

The young woman in such cases is called awkward, and, perhaps is compelled to sit up in bed before the third day, in order to hold the child better. The medical man should give personal attention to this procedure, unless he is certain there are attendants who will make it a matter of conscientious duty. The contraction of the uterus which attends the first drawing of the breasts, is an important fact, and is a potent reason for attending to it. In these days of uterine ailments, it is very important that every encouragement should be afforded to involution. Formerly it was urged as a reason for allowing the child to take the breast early, that the first few drains of milk contained a cathartic principle. While that is admitted, the availing ourselves of an important excito-motory phenominon in order that the womb may as soon as possible regain its unimpregnated dimensions, is of greater importance.

For years, I have with much delight, noticed the peculiar facial expression commonly known as a "scowl," when a woman has her child at the brest. Perhaps every one is not happily favored with that reflex sensitioness which is supposed to exist between the organs so curiously associated in welfare of a new being.

Do we not find a fair proportion of our cases of involution, among that class who prefer to fondle a poodle dog, to the nursing of their own children?

Returning from a brief digression, we may devote a few thoughts to a common source of anxiety to the matrons, if not the physician, viz: A failure to urinate.

Very often a child will have emptied the rectum long before any sign of urinating will be seen. When we consider that the kidneys have at birth but just felt called upon to exercise their peculiar function, a little time should be allowed for secretory work to be accomplished. Upon the first call made after labor is finished, the question should always be asked, Has the child urinated? If not, and we have waited a reasonable time, a teaspoonful of castor oil may be given, which, with its operation, will be very certain to have associated, a free discharge of urine.

In this connection, the idea is suggested that in a great majority of cases when we wish to promote renal activity, that the best *diurectic*, is a judiciously selected *cathartic*.

There is another subject which may be introduced here, that is, the influence of the mother's milk upon her infant. The importance of properly evacuating the lying-in woman's bowels before the third day is appreciated by all observers. The normal action of the rectum has been disturbed during the later months of pregnancy, and fecal accumulations are apt to be found after delivery, unless strict attention is paid to this, there will be fever at the third day, (milk fever commonly called, but questioned by some as to its being an necessary concomitant) the milk more abundant than it otherwise would be, and holding much morbid matter, which will prove injurious to the child. There is no doubt but that many infants snffer as well as the mother from the want of a prompt removal of all morbid and fecal matter within thirty-six hours.

When called upon to advise in cases of infantile ailments during the first weeks from birth, I make it an unvariable rule to ascertain the true condition of the mother, and very frequently find my apprehensions arê well founded. It is bad treatment to permit the mammary glands to do vicarious work in elimination of *effete matter.*

Besides being the receptacle of morbid matter which will injure an infant, the milk is very often affected by the mental condition of a woman, or perhaps more properly, the child receives impressions upon its nervous system through the lacteal secretion. Cross children have cross mothers, and many an infant is restless and excitable during the night after its mother has, during the previous day, worked hard at some fatiguing employment, or indulged in a round of pleasure or dissipation. In those sections of our country where women perform most of their own domestic work, this fact is very apparent.

I am in the habit of relating the following case as illustrating this subject.

During the summer of 1854, it was my sad lot to witness the ravages of Asiatic cholera. Its ravages were not then, as during the past season (1873), confined to the lower and poorer classes. It invaded the ranks of the rich and educated. Among the incidents of the time, was the sickening of a talented lawyer, his lovely wife, and two little girls. During the night in which the parents both died, and the children required unremitting attention, a lady friend of the family, of warm attachments and tender sensibilities, remained till early dawn. Depressed by grief, fatigued and alarmed for her own safety, she went home and at once nursed her infant boy of less than eighteen months. In a few minutes the child began to vomit, and void from the bowels very copiously of a watery secretion. It had been well on the day before, and showed no signs of choleraic disease during the night. I was at once called, and from my knowledge of all the facts, stated that the trouble all arose from the mother's mental condition.

So sure did I feel of the correctness of my views, that nothing was advised by way of medicine, but the breast must be withheld. Within a few hours, the child was free from the unpleasant symptoms, and the next day was as bright as ever.

Doubtless every practitioner of experince has known similar instances.

